# GEUINF: Real-Time Visualization of Indoor Facilities Using Mixed Reality

**DOI:** 10.3390/s21041123

**Published:** 2021-02-05

**Authors:** David Jurado, Juan M. Jurado, Lidia Ortega, Francisco R. Feito

**Affiliations:** 1 Computing and Numerical Analysis Department, University of Córdoba, 14071 Córdoba, Spain; 2 Computer Graphics and Geomatics Group of Jaén, University of Jaén, 23071 Jaén, Spain; jjurado@ujaen.es (J.M.J.); lidia@ujaen.es (L.O.); ffeito@ujaen.es (F.R.F.)

**Keywords:** mixed reality, facility management, real-world scanning, geometric alignment, ARCore

## Abstract

Mixed reality (MR) enables a novel way to visualize virtual objects on real scenarios considering physical constraints. This technology arises with other significant advances in the field of sensors fusion for human-centric 3D capturing. Recent advances for scanning the user environment, real-time visualization and 3D vision using ubiquitous systems like smartphones allow us to capture 3D data from the real world. In this paper, a disruptive application for assessing the status of indoor infrastructures is proposed. The installation and maintenance of hidden facilities such as water pipes, electrical lines and air conditioning tubes, which are usually occluded behind the wall, supposes tedious and inefficient tasks. Most of these infrastructures are digitized but they cannot be visualized onsite. In this research, we focused on the development of a new application (GEUINF) to be launched on smartphones that are capable of capturing 3D data of the real world by depth sensing. This information is relevant to determine the user position and orientation. Although previous approaches used fixed markers for this purpose, our application enables the estimation of both parameters with a centimeter accuracy without them. This novelty is possible since our method is based on a matching process between reconstructed walls of the real world and 3D planes of the replicated world in a virtual environment. Our markerless approach is based on scanning planar surfaces of the user environment and then, these are geometrically aligned with their corresponding virtual 3D entities. In a preprocessing phase, the 2D CAD geometry available from an architectural project is used to generate 3D models of an indoor building structure. In real time, these virtual elements are tracked with the real ones modeled by using ARCore library. Once the alignment between virtual and real worlds is done, the application enables the visualization, navigation and interaction with the virtual facility networks in real-time. Thus, our method may be used by private companies and public institutions responsible of the indoor facilities management and also may be integrated with other applications focused on indoor navigation.

## 1. Introduction

Mixed reality (MR) merges real and virtual world elements giving the sensation that they coexist in the same environment. Regarding augmented reality (AR), MR adds knowledge about the physical surrounding world allowing a better integration and immersive experiences. MR is considered a technological innovation that has been incorporated into traditional disciplines such as architecture or engineering. Its benefits and opportunities are encouraging and currently under study [1]. From the design and modeling phases to the construction or development, teams of professionals involved can benefit from MR [2,3]. Guidance is a constant in many of these applications, regardless of the objectives. This goes from task support in industrial environments [4] or the need of users to orientate and find paths into buildings [5,6]. Another example is to guide technicians in complex maintenance tasks, improving the life cycle of buildings and infrastructure [7].

One of the reasons why MR is still considered a research topic for facility management is the need of an accurate alignment between real and virtual worlds. This issue is not really relevant in some AR applications, in which no physical constraints are considered and virtual objects can be placed anywhere, even floating in the air. MR goes one step further, both worlds must be correctly overlapped considering a spatial consistency [8]. This implies the understanding of the user environment and a optimal adjustment in order to locate each virtual model, which represents every indoor facility, where it has been fixed considering both for the design and construction phase and maintenance phase. To establish mentioned matching, it is mandatory to know the user orientation and position in the real world. In this way, previous work is proposed related to indoor navigation and positioning [9,10,11,12].

Initially, the process of achieving this geometric fit often required sophisticated devices and techniques based on computer vision. In fact, significant contributions have been proposed according to the use of a set of sensors are integrated to obtain an accurate user position and orientation such as the inertial measurement unit (IMU), the Global Positioning System (GPS) and the radio frequency identification (RFID) [13,14]. More recently, AR has benefited from advances in sensing hardware that is embedded in mobile devices. Additionally, Application Programming Interfaces (API) are being developed for their integration for useful applications focused on the representation of virtual 3D models in outdoor real-world scenarios [15].

Enabling MR indoor implies different techniques or even advanced hardware for positioning, since GPS is usually not available. Most of the proposed methods for the pose tracking are based on using physical markers fixed around the user environment. A marker is a pattern with an unique identifier widely represented using QR codes. These can be detected applying homography-based visual tracking for two main objectives: (1) to determine the position and orientation of the user camera and (2) to fix the position of some virtual objects. So far, this has been one of the most widely used methods to find paths and infrastructures [16]. Even when positioning and orientation can be considered solved with the use of markers, the problem remains when free navigation is enabled [17]. Although tracking and sensing technologies are able to estimate the initial position and orientation, both must be corrected during the navigation process. The solution requires additional markers in the real scenario in order to pose correctly the virtual camera in the real world. The generation and placement of these human-made markers involve a time-consuming task for large places, as well as multiple limitations regarding the automation of the process.

In order to solve the inaccuracy of GPS to determine the user position into indoor scenarios, the Internet of Things (IoT) may be an alternative since significant data can be obtained from the real world [18,19,20]. However, it implies the production of a sophisticated sensor system that can be plausible mainly for functional industrial environments, but its accessibility is lower in contrast to smartphones which are the most widely used device around the world. In the literature, several approaches are exposed for indoor positioning based on Wifi technology, but the resulting location is not accurate enough and the error can be close to a few meters in some places where the signal is low [21]. Nevertheless, the estimation of the user position in indoor scenarios is not the only challenge. In addition, the user camera orientation has to be estimated and both must be updated during the user navigation.

Our markerless-based approach implies a significant advance for the use of mixed reality using geometrical data scanned from the user environment in order to know both the user location and orientation in indoor scenarios [8]. In this regard, there have been previous works focused on the removal of physical markers but with the drawback that additional sensing such as radio frequency identification, laser pointing and motion tracking must be added [22]. Other approaches have presented several contributions to detect objects or key points of a scene by scanning the environmental features and thus, obtaining a 3D map. Most of these techniques are based on Simultaneous Localization And Mapping (SLAM) in order to estimate the pose of a moving monocular camera in unknown environments [23]. Among these techniques, RGB-D sensors are widely used since these are cost-effective devices able to capture colorful and depth images [9,24,25]. However, the methodology based only on RGB-D sensors may be affected by inconsistencies between depth data and color images. Therefore, some approaches take advantage of the fact that most indoor environments follow the Manhattan World assumption, in which the spatial layout constraints facilitate the camera tracking [12,26,27]. Even so, some authors ensure that accuracy during free navigation must be checked and adjusted, for instance, by planar surface matching [28] or by means of video frames [12,26].

In the field of architecture, the most remarkable MR contribution is related to the representation of a virtual model at a full scale in order to visualize the design onsite [2]. Building Information Modeling (BIM) or Computer-Aided Design (CAD) are the most used software tools for design in architecture and engineering projects [29]. CAD has been used in architecture since the 1980s and these tools are still very common in a building design [30]. However, the main advantage of BIM regarding CAD is going beyond the planning and design phase, extending throughout the whole life cycle and allowing access to all agents involved [7], while improving risk identification in all phases of the project [31]. In any case, these tools and data formats are not directly transportable to the construction site by means of mobile devices. Tablets or smartphones are still far from being part of the infrastructure monitoring process, and therefore they do not contribute to their life cycle. In this regard, literature points to AR and MR as novel technologies that can make BIM/CAD ubiquitous [5,32]. In this sense, the development of markerless techniques is highly demanded to complement BIM-based design [33]. Thus, structural elements of buildings under construction can be identified by overlapping augmented 3D models on the real-world scenario and even on the design plan. From the early stages of construction, buildings change continuously, so the use of fixed markers is not a valid solution [34]. However, if no markers are used for the environment recognition, the use of other meaningful data have to considered. In this type of scenarios, the only permanent elements from the early stages of construction are structural components such as pillars, walls or floors. These planar surfaces can be compared with the ones tracked in real time using the depth information provided by the most recent smartphones [25,35]. Likewise, in the phase of maintenance, the building structure does not change, and a geometrical comparison between normal vectors of tracked and virtual walls can be performed in real time in order to recognize the user environment [36].

According to the facility management is a key topic that is ultimately addressed by augmented and mixed reality, although, traditionally, it has been carried out with sophisticated sensing systems [37,38]. However, this process can benefit from the geometric correspondence between architectural planes and the resulting structure after its construction [39,40]. BIM and AR used together are increasingly considered as the paradigm for decision making during the construction [3] and to ease facility management throughout its life cycle [41]. MR adds an additional advantage during maintenance allowing visualization and interaction with facilities hidden behind walls or floors [42], as well as asphalt [15].

In this paper we propose GEUINF (Geospatial and Environmental tools of University of Jaén—Infrastructures), a new module of GEU developed by Jurado et al. [43]. GEU is a novel framework which is formed by several applications focused on different research fields like computer graphics, computer vision and remote sensing. In this work, we present GEUINF which is based on a markerless approach to enhance the visualization of indoor facilities using mixed reality. In this way, geometrical models of a CAD architectural project may be represented as augmented 3D entities into the user environment in the real world. This alignment between real and virtual models is based on planar surface detection which are directly scanned from the user environment in the real world. Once this alignment is performed, we can register the user position and orientation in real time and show the hidden facilities using ubiquitous systems. This alignment between real and virtual environments is achieved by preprocessing the original 2D planes from the CAD project in order to obtain a topological 3D representation of its architectural elements. As a result, indoor facilities, whose inspection is quite difficult since these are not directly visible, can be reviewed onsite using our solution.

## 2. Methods

**Figure 1:**
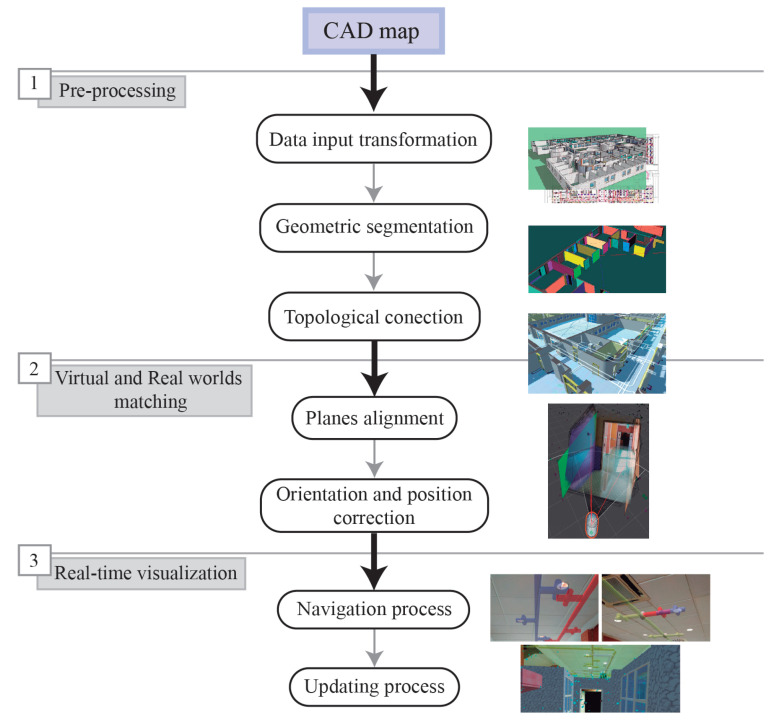
The flow diagram of the proposed methodology.

In this section, the algorithms developed to scan, process, and visualize indoor infrastructure as 3D augmented models on the user environment are presented. The key idea is to detect in real-time the user position and orientation on one floor of the surveyed building by scanning the user environment and comparing it with its replicated virtual scenario. Thus, each part of infrastructure becomes visible in mixed reality after the user environment has been recognized. Then, it is possible to enable a real-time visualization of hidden facilities, which can be inspected onsite by an intuitive way using portable devices. The methodology is based on three main stages: (1) preprocessing input data, (2) real-world scanning and matching of virtual and real worlds and (3) real-time visualization in mixed reality. Figure 1 presents the main steps of the proposed methodology.

In the first step, we start from the 3D model of a building floor obtained from the 2D CAD project. This information includes some structural elements such as walls and pillars. Later, this 3d model is processed to obtain wall sections topologically connected. These segmented walls are used to be compared with the physical environment, assuming that the building structure is enough to determine the user position and orientation in the virtual world.

In the second phase, virtual and physical worlds are matched by aligning both cameras in terms of orientation and position. To this end, the real world is scanned using the ARCore library which is supported by most portable devices. This library enables the modeling of horizontal and vertical surfaces of the user environment. Then, the information obtained from the physical world is compared with the one from the virtual world, generated in the preprocessing stage. The geometrical alignment is still done through geometric comparison, but now the search for candidate walls is reduced thanks to the topological configuration of the data model. The resulting transformation matrix after applying the mentioned aligned is used to correct the initial orientation provided by the mobile compass that presents an angular mishmash between real and virtual planes. After understanding the user environment in the virtual, the position is determined by applying a distance-based computation from the initial user position to the surrounding elements.

The third phase occurs once both worlds match and are aligned. Then, navigation and visualization of hidden infrastructures can be enabled using a friendly user interface which describes the status and features of the observed facilities. Free navigation is possible avoiding markers by taking advances of the spatial coherence with the virtual topological virtual model. The user position and orientation is updated in real time by comparing the new scanned planes with the virtual model over time. Moreover, the user can interact with augmented models by easy-to-use gestures on the screen.

GEUINF has been developed in C# using the cross-platform game engine Unity, which is able to integrate all ARCore functions and adds a user-friendly interface. For testing an smartphone (model: Google Pixel XL) is used.

### 2.1. Preprocessing of Data

The first step of the proposed methodology is the preprocessing of input data. In this section, the digitization of architectural components and target infrastructures of the building is described. The goal is to generate a 3D topological model in a virtual scenario that represents most features that belong to the study area. Thus, this section is divided in three main parts (1) input data transformation, (2) triangle connection and wall segmentation and (3) definition of topology between wall sections.

#### 2.1.1. Input Data Transformation

**Figure 2:**
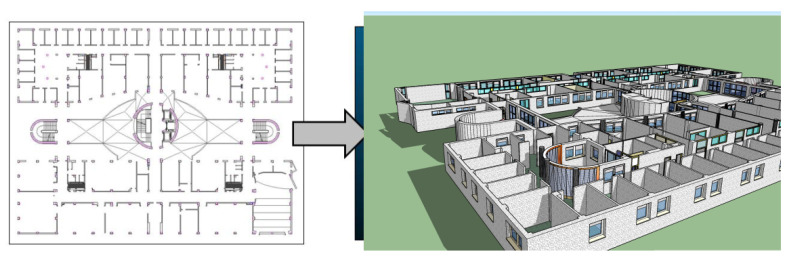
The resulting 3D model from 2D CAD layers.

In this research, the dataset used as input data is provided by the university’s polytechnic school of Jaén. We focus on the first floor of this building, in which teaching offices and laboratories are located. This building is already available in 3D since Dominguez et al. [44] developed an efficient method to generate 3D virtual data of our study area. This process is based on transforming every information layer from a 2D CAD map to a 3D model enriched with furniture and indoor infrastructures. Initially, this method selects some layers corresponding to architectural elements such as walls or pillars using MapInfo [45]. Then, these 2D lines are converted to 3D walls, providing surfaces of walls, ceilings and floors. This semiautomatic process is performed using SketchUp software [46], which helps to obtain visually relevant results in the architectural field, as depicted in Figure 2.

**Figure 3:**
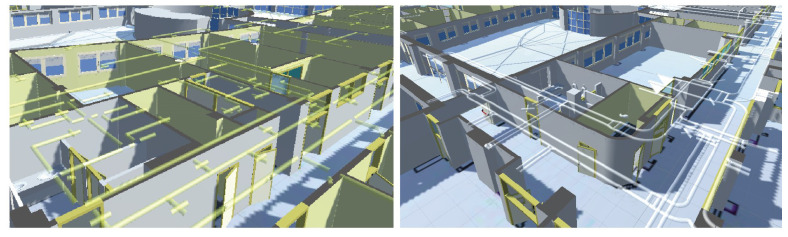
Virtual representation of indoor infrastructures and structural elements of the study area.

In addition, the 3D model is enhanced adding additional 3D structures related to facilities of water, air conditioning, electricity or ventilation. The resulting virtual environment is shown in Figure 3. There is a requirement in the way that these facilities are included since they must preserve scale and position regarding the structural elements. Therefore, input lines representing wires and pipes must be superseded by 3D models and placed in the correct positions according to the original plans. This 3D model is a mesh of unlinked triangles without any topology. In this sense, the topology that must be defined in order to perform an efficient search of candidate planes to be compared with scanned walls in the real world. Consequently, the next step is to develop a geometric segmentation in order to convert every wall into multiple 3D sections connected to each other.

#### 2.1.2. Topological Segmentation of Virtual 3D Model

Once the virtual representation of the building floor was obtained, the following step was to add topological relations to each triangle and to perform wall segmentation. This implies to separate the geometry of walls into sections, which restricts the search scope for planar surfaces and accelerates the alignment process. A wall section is considered a set of connected triangles matching a single plane. Then, these triangles must be adjacent and have the same normal vector. Therefore, the method described in this section puts together annexed triangles with the same orientation to construct a wall section. Therefore, a wall section is represented by a unique normal vector, which will be compared with the normal vector of those flat surfaces tracked in real time with ARCore. We rely on the premise that facility networks are spatially linked to virtual walls in the original CAD and that these constraints are maintained in 3D. Therefore, when real and virtual wall planes line up, facilities are accurately placed, as observed in Section 4.

**Figure 4:**
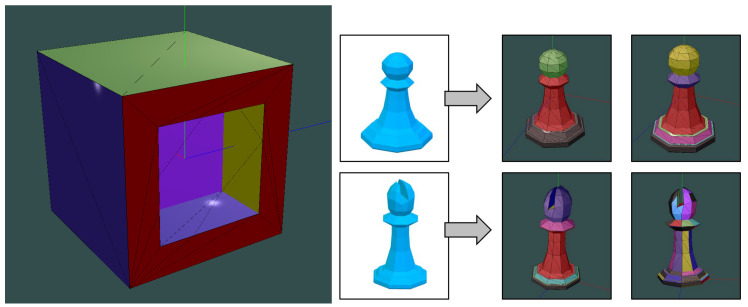
Training dataset to fit our method for coplanarity-based segmentation.

The proposed segmentation method has been tested with a training dataset as shown in Figure 4. Using diverse colors, the cube is separated by the different planes containing their faces. In this case, planes associated with these faces are well defined because neighboring faces are orthogonal. However, as some walls in the building are curved, a coplanarity factor has to be defined in order to determine those walls that take part of the same section. The right image on Figure 4 shows the results by changing the value of the coplanarity factor in chess figure models.

The input 3D model of the study area is not characterized with a geometrical topology. This only contains multiple triangles that compose the surface of 3D objects without a semantic meaning. In this work, we propose a relevant advance in this field by connecting neighboring triangles in order to create wall sections as mentioned before. This topology layer enables a significant acceleration to compare scanning data from the real world with the virtual 3D model. Initially, they are arranged as nonconnected triangles or triangle mesh (see Figure 1). This means that a triangle does not know which are its three neighboring triangles. In order to connect all of them topologically, an exhaustive algorithm starts from an arbitrary triangle considered as seed. This triangle is assigned an identifier (a specific color for its representation). Then, its three neighboring triangles are searched. When one of them is found, the same color or identifier is assigned only if both triangles are coplanar considering a specific coplanarity factor. For this, their normal vectors must be equal or similar. Thus, both triangles are topologically connected as part of the same wall section.

**Figure 5:**
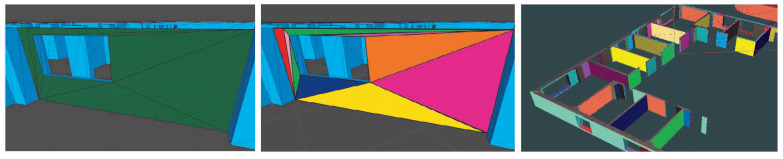
Generation of wall sections by connecting coplanar triangles.

If the triangle is a neighbor but not coplanar, and not yet assigned a color, it is considered a seed triangle of a new wall section. The process uses the classic MFSet (Merge-Find Set) data structure [47], which allows one to perform this search as efficiently as finding paths in graphs. Figure 5 shows several nonconnected triangles resulting from the 3D modeling process. Once segmentation is applied to the whole building, we obtain the set of wall sections depicted in Figure 5. When each wall section is identified with different color or identifier, then the matching procedure with the real world is facilitated. This way, the yellow triangle in Figure 5 is topologically connected with the blue and pink triangles and they are part of the same wall section. Each wall section has the same normal vector, which will be compared with the normal vector of planar surfaces scanned from the real world.

Once triangles in the same wall section are topologically connected, the last process also consists of adding new topological relations between nearby wall sections. This relationship between wall sections helps to find the building geometry to be compared with the scanned planes from the real world. The result is a real-time matching between virtual and real worlds. In the right image of Figure 5, any wall section is also connected to its adjacent wall section, normally with $90^{\circ }$ except in curved walls. Once preprocessing stage is completed, the next step is to recognize the real world by scanning the user environment and comparing with the virtual 3D scenario.

### 2.2. Real-World Scanning

In this section, the acquisition process in order to capture 3D data from the real world is described. The proliferation of new smartphones, whose RGB camera integrates two or more lenses, enables to reconstruct 3D model of the user environment. In fact, recent studies use ARCore library to explore cultural heritage [48], to develop a navigation system for visually impaired people [49] and other computer vision applications [50]. In this study, we used portable devices as smartphones due to the high accessibility and their capabilities to capture real-world data and render virtual models using MR.

**Figure 6:**
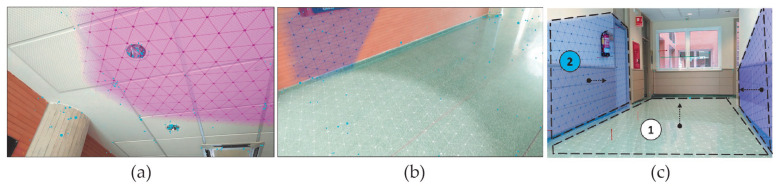
Plane surface tracking using ARCore: (**a**) horizontal surface scanning, (**b**) vertical surface scanning and (**c**) calculation of normals.

The first step of the proposed methodology is to scan the user environment in order to determine the user position and orientation in the virtual world, which includes a 3D model of the building and service infrastructures as mentioned in Section 2.1. For this purpose, Google ARCore is used to detect and model surrounding walls of the user position. This API is based on SLAM to estimate the pose of the device regarding the world around it. In contrast to other studies based on RGB-D cameras for 3D reconstruction [51], ARCore only needs a RGB camera to identify 3D feature points from overlapping frames obtained while the user is walking around the building. In this study, the user must establish its initial position $p_{v}$ into the virtual environment by clicking on a specific location into the 2D representation of the virtual world. Obviously, $p_{v}$ is approximate with respect to its current position in the real world, but the associated errors are later corrected by our method. Regarding camera orientation in the virtual world, it is initially fixed with the orientation provided by the device compass. After that, the real world is scanned with ARCore and a set of planes are detected while the user walks with the device. The resulting 3D planes are stored as $AR_{w}=\left\{aw_{1},aw_{2},...,a_{wk}\right\}$. ARCore focuses on searching clusters of feature points to model both horizontal and vertical surfaces represented as 3D planes. In this way, during a few seconds, our goal is to capture planes close to the observer, like walls, ceiling and floor and to obtain its geometry from feature points detected in the real world (Figure 6). Only when the device estimates that the plane recognition is accurate, it provides and visualizes a mesh of dots and triangles that adjusts with a high degree of precision to the surface that is being scanned, as the figure reflects. ARCore is very accurate once the planar surface is detected. This technology has no problems detecting most surface types, although it works faster with rough textured surfaces and changing colors. For example, posters on walls are more easily detected than plain surfaces. Regarding distances, ARCore is able to detect surfaces up to a distance of 5 m. So that, in the real environment where our tests have been carried out, all these circumstances have been given correctly, as described below.

After capturing real-world data, the next significant contribution of this research is to estimate the user orientation in real time by scanning the user environment. The method to match the detected planes with their corresponding virtual planes and the alignment process is described in Section 2.3.

### 2.3. Matching of Virtual and Real Worlds

**Figure 7:**
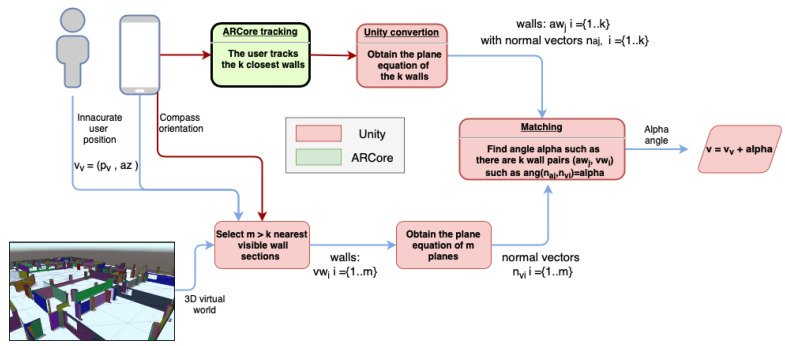
Matching wall process using two walls and orientation correction.

The use of mixed reality to visualize indoor infrastructure of the virtual 3D model requires a full alignment between the virtual and real worlds. In this section we describe how to match both environments, the virtual and real worlds. Firstly, we describe Algorithm 1 for matching the detected walls with ARCore with their twin wall sections in the virtual model. Then, two geometric transformations (translation and rotation) are applied in order to correct the orientation and position of the user camera in the virtual world (Algorithm 1). Thus, we ensure a correct position and orientation of all virtual infrastructures to be visualized in our application using mixed reality. An overview of this method is shown in Figure 7.

**Figure 8:**
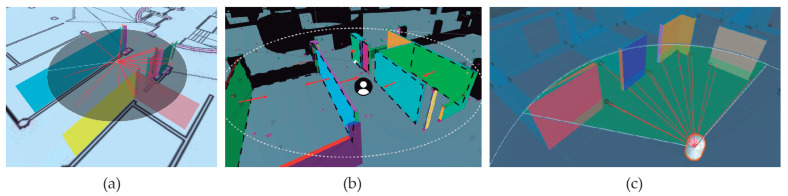
The selection of surrounding walls to the user position in the virtual world: (**a**) Plane selection in the search area, (**b**) Normal calculation of virtual planes and (**c**) computing of plane visibility from the user view.

Once *k* real walls are scanned with ARCore, as described in Section 2.2, the following step is to search for their corresponding virtual walls. For that purpose, the first step is to search walls in both environments, as Algorithm 2 describes. According to the position fixed by the user in the virtual world $p_{v}$, a search of surrounding walls is performed. The stopping criteria for this research is fixed when the number of *m* visible planes is equal or higher than *k* scanned planes in the real world. The plane proximity is established by distance calculation. Figure 8a shows those planes which are into the search area. As a result, an array of several virtual planes, $VR_{w}=\left\{vw_{1},vw_{2},...,vw_{m}\right\}$ is obtained, m≥k with walls from the virtual world. All walls stored in $AR_{w}$ are also contained in $VR_{w}$. A wall $vw_{i}\in VR_{w}$ is visible from $p_{v}$ if the dot product of its normal vector $n_{vi}$ and the vector with origin in the wall $vw_{i}$ and end in $p_{v}$ is greater than 0. Additionally, the ray starting from $p_{v}$ that reaches each $vw_{i}$ does not intersect any other wall (see Figure 8b). Finally, Figure 8c shows the selected section of walls that are visible from $p_{v}$. Therefore, Algorithm 2 (1) obtains a set of *k* real walls $AR_{w}$, and (2) find m≥k visible virtual walls, $VR_{w}$, around the user position $p_{v}$.


**Algorithm 1:** Matching between virtual and scanned scenarios.

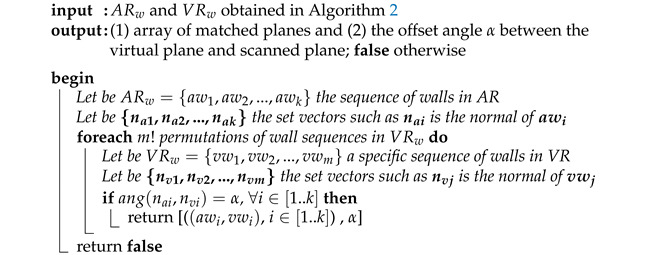




**Figure 9:**
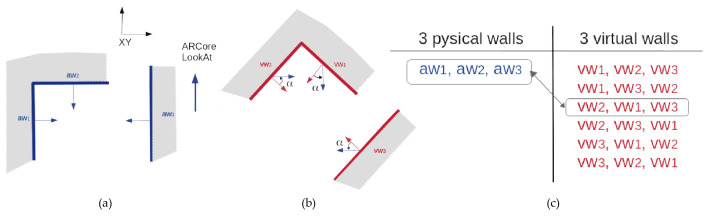
Angular offset α: (**a**) the scanned planes from real world, (**b**) the corresponding planes of the virtual world (red) and (**c**) pairs of real and virtual walls to be compared.

The next step is to match planes of AR${}_{w}$ and VR${}_{w}$. This task is necessary since both sets of planes are not aligned due to the mobile compass inaccuracy up to around 12 degrees, according to our validation process. Consequently, a mismatch angle α around Z-axis is presented between scanned and virtual scenarios. Figure 9 shows a visual representation of this rotation where the virtual world (red) is rotated at an α angle respected to the real world (blue).

Algorithm 1 describes the main steps for matching real and virtual worlds. The input data are the wall sets resulting by applying Algorithm 2. The output is twofold: (1) a correspondence between virtual and scanned planes and (2) the offset angle (α) between matched planes. The proposed method is based on calculating the angle between normal vectors of virtual and scanned planes.
**Algorithm 2:** Extraction of virtual planes to be matched with scanned planes from the real world.
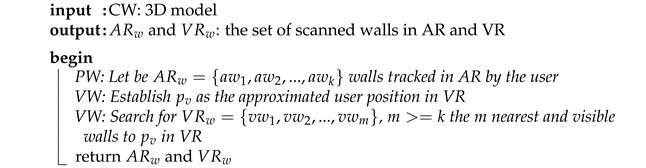


In order to find this offset angle, we should find which wall $aw_{i}$ in the physical world matches with the wall $vw_{j}$ in the virtual. Thus, for a given sequence of walls in ${\mathrm{AR}}_{w}$, we should find the sequence of k walls in ${\mathrm{VR}}_{w}$ such that each pairwise $\left({\mathrm{aw}}_{i}-{\mathrm{vw}}_{i}\right)$ matches the real wall ${\mathrm{aw}}_{i}$ with the virtual one ${\mathrm{vw}}_{i}$. If we fix the sequence of k walls in AR as ${\mathrm{AR}}_{w}=\left\{{\mathrm{aw}}_{1},{\mathrm{aw}}_{2},...,{\mathrm{aw}}_{k}\right\}$ (step 2), we must check which of the m! permutations of ${\mathrm{VR}}_{w}$ gives the same wall sequence. We know that m≥k, so the sequence of ${\mathrm{AR}}_{w}$ of size k is contained into this set of m! combinations of walls in ${\mathrm{VR}}_{w}=\left\{{\mathrm{vw}}_{1},{\mathrm{vw}}_{2},...,{\mathrm{vw}}_{m}\right\}$. Therefore, we need to chek only the first k elements in a sequence of ${\mathrm{VR}}_{w}$. As pairwise wall comparison is done by means of their normal vectors, we compute $\left\{n_{a1},n_{a2},...,n_{\mathrm{ak}}\right\}$ as the corresponding sequence of normal vectors for ${\mathrm{AR}}_{w}$ (step 3). The **foreach** loop checks which of the m! sequences of normal vectors associated to ${\mathrm{VR}}_{w}$ corresponds with the given sequence of normal vectors of ${\mathrm{AR}}_{w}$. Then, each iteration compares if the offset angle of each pairwise is always α (steps 5–10). In fact, the angle is not necessary to be computed. It is enough to calculate the cosine from the dot product formulation. Then, we compute $\cos\left(\alpha \right)=\left(n_{\mathrm{ai}}\cdot n_{\mathrm{vi}}\right)/\left(|n_{\mathrm{ai}}\left|\right|n_{\mathrm{vi}}|\right)$, to compare this α angle candidate with the rest of pair candidates $\left(n_{\mathrm{vi}},n_{\mathrm{wi}}\right)$, i=1,..,k. Considering our case studies, we observe the number of walls may be sufficient with two or three well-defined walls. An example of this search is depicted in Figure 9c considering k=3 and m=3 walls. Starting from the specific sequence of walls in the physical world, $\left({\mathrm{aw}}_{1},{\mathrm{aw}}_{2},{\mathrm{aw}}_{3}\right)$, we should find which of the m=3!=6 different sequences of walls in ${\mathrm{VR}}_{w}$ provides this matching. In the example, sequences $\left({\mathrm{aw}}_{1},{\mathrm{aw}}_{2},{\mathrm{aw}}_{3}\right)$ produce the same α angle when comparing with the sequence $\left({\mathrm{vw}}_{2},{\mathrm{vw}}_{1},{\mathrm{vw}}_{3}\right)$. This means that the result of Algorithm 1: $\left({\mathrm{aw}}_{1}-{\mathrm{vw}}_{2}\right)$, $\left({\mathrm{aw}}_{2}-{\mathrm{vw}}_{1}\right)$ and $\left({\mathrm{aw}}_{3}-{\mathrm{vw}}_{3}\right)$.

### 2.4. Correction of the User Position and Orientation

In this section, once the both virtual and real worlds are aligned, a geometric transformation must be applied to overlap and display the virtual infrastructures on their correct position and orientation. To this end, the camera of the virtual world is set considering two two main steps: (1) the correction of orientation angles and (2) the correction of the 3D position.

According to the output of the proposed method for matching real and virtual worlds, an angle α is estimated. As mentioned in Section 2.2, this angle is used to rotate the virtual camera around Z-axis applying Equation (1). Thus, the error caused by IMU’s device is corrected by checking the user environment in real time (see Figure 7).

(1) $$R=\left(\begin{array}{ccc} \cos\alpha & -\sin\alpha & 0\\ \sin\alpha & \cos\alpha & 0\\ 0 & 0 & 1 \end{array}\right)$$

where α is the angle offset between virtual planes and their corresponding scanned planes in the real world.

**Figure 10:**
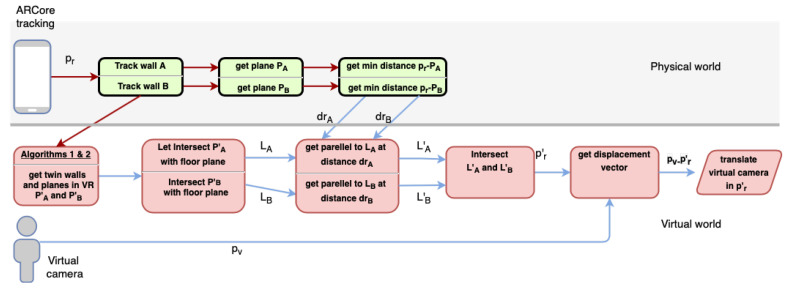
Workflow to correct the user position in the virtual world.

After the orientation correction, the next step is to do the same with the virtual camera position, that is, to adjust the user position in the virtual world. The scheme in Figure 10 summarizes this process.

**Figure 11:**
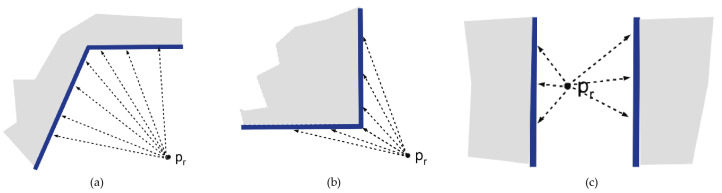
The user is located in $p_{r}$ close to a couple of walls: (**a**) inner corner, (**b**) outer corner and (**c**) parallel walls.

The goal of this method is to correct the user position in the virtual world by applying a translation vector which is calculated based on a measurement of distances from the user position to the closest 3D planes (walls in the real world). The user is located in a position $p_{r}$ (point in the real world), where these few walls are visible. The device is able to track adjoining walls, making an inner corner for example as in Figure 11b. Another valid position is the outer corner in Figure 11a. Starting in a hallway, between parallel walls as in Figure 11c, involves more difficulty in determining the initial position. This situation, however, is valid for preserving alignment during free navigation, as stated in Section 4.

**Figure 12:**
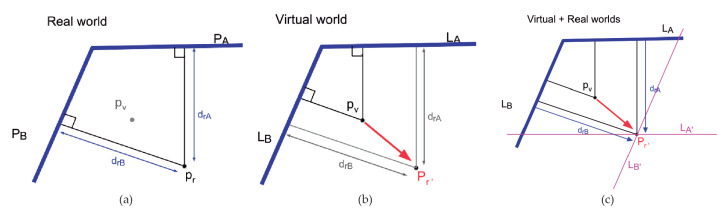
Process for obtaining the displacement vector $p_{v}p_{r}$: (**a**) the real world, (**b**) the virtual world and (**c**) the translation vector.

As stated before, from the real world observed with ARCore, we can obtain real distance measurements. To compute minimal distance from $p_{r}$ to any of these walls, we first get the equation for the plane $P_{A}$ and $P_{B}$ from the set of points in the wall obtained by ARCore during the tracking process. Figure 12 shows this situation with any inner corner, even with nonorthogonal walls.

The parametric equation of the plane can be obtained from three points $p_{1}$, $p_{2}$, $p_{3}$ belonging to this plane. Our scanning method only captures plane surfaces when the surface is sufficiently representative. When these planes are acquired, a set of representative surface points in each frame (acquirePointCloud()) can be used to define the Plane equation, as shown in Table 1 that contains the plane equation and the mathematical representation of the normal vector. Then, minimal distances are computed $d_{\mathrm{rA}}$ and $d_{\mathrm{rB}}$ from $p_{r}$ to planes $P_{A}$ and $P_{B}$ respectively. Focusing on the distance of $p_{r}=\left(x_{r},y_{r},z_{r}\right)$ to the plane $P_{A}$, we compute $d_{\mathrm{rA}}$ by using formation presented in Table 1.

Once real distances $d_{\mathrm{rA}}$ and $d_{\mathrm{rB}}$ are obtained, then we focus on the virtual world. We know $p_{r}$ in the ARCore reference system but not $p_{r}^{\prime }$ in the virtual one. The user estimates that they are in $p_{v}$, but there can be several error meters from $p_{r}^{\prime }$. Then, $p_{r}^{\prime }$ is the exact position of the user camera in the virtual reference system to match physical and virtual worlds.

Planes $P_{A}$ and $P_{B}$ are already known in VR as $P_{A}^{\prime }$ and $P_{B}^{\prime }$ after matching walls when running the method of Algorithm 1. However, we do not need to work in 3D at this level. Walls are known to be vertical, and distances can be calculated in 2D, so that we consider $L_{A}$ and $L_{B}$ as the resulting lines of intersecting planes $P_{A}^{\prime }$ and $P_{B}^{\prime }$ with the floor plan (see Figure 12b). $L_{A}$ and $L_{B}$ are given as the parametric form $L_{A}=a+t\;\vec{\mathrm{ac}}$ and $L_{B}=b+s\;\vec{\mathrm{bd}}$ for any points a and c in $L_{A}$, and b and d in $L_{B}$. We need to compute vector $v=p_{v}p_{r}^{\prime }$ to displace the virtual camera from $p_{v}$ to the correct position $p_{r}^{\prime }$. Then, $p_{r}^{\prime }$ is computed as the intersecting point of lines $L_{A}^{\prime }$ and $L_{B}^{\prime }$, as depicted in Figure 12c. $L_{A}^{\prime }$ is the parallel line to $L_{A}$ at distance $d_{\mathrm{rA}}$. We compute $L_{A}^{\prime }$ as the line $L_{A}^{\prime }=a^{\prime }+t\;\vec{\mathrm{ac}}$, that is, the same direction of $L_{A}$ but going through point $a^{\prime }$ at the distance $d_{\mathrm{rA}}$ from point a (see Table 1). Similarly, $L_{B}^{\prime }$ is obtained to distance $d_{\mathrm{rB}}$ from $L_{B}$. The intersection point $p_{r}$ is obtained by solving the line equation $L_{B}^{\prime }$ for the s value. The resulting points $p_{r}$ and $p_{r}^{\prime }$ are now equivalent and both realities coincide regarding scale, orientation and position.

## 3. Validation and Accuracy Assessment

**Figure 13:**
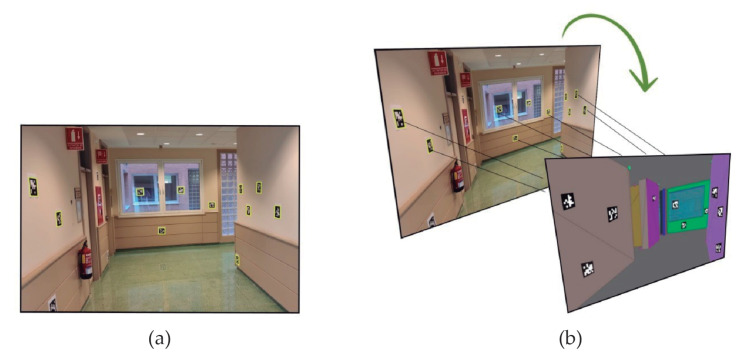
Validation process using fixed markers to validate the user position and orientation: (**a**) marker detection and (**b**) estimation of corresponding anchors in the virtual world.

ARCore reliability has been tested in order to validate the technology to avoid markers. The validation process has been developed following these criteria: (1) the initial position in the virtual world is fixed by the user and (2) the initial orientation is estimated taking as reference several fiducial squared markers with unique identifications, which are fixed with the same position both in real and virtual worlds. The marker detection allows us to accurately estimate the initial user orientation in the real world. While the user starts scanning the surrounding walls, several markers are detected (Figure 13a) and these are automatically instantiated in the virtual world as new anchors, see Figure 13b. Finally, a geometry transformation (rotation and translation) is computed to align old virtual anchors with their corresponding new anchors.

**Figure 14:**
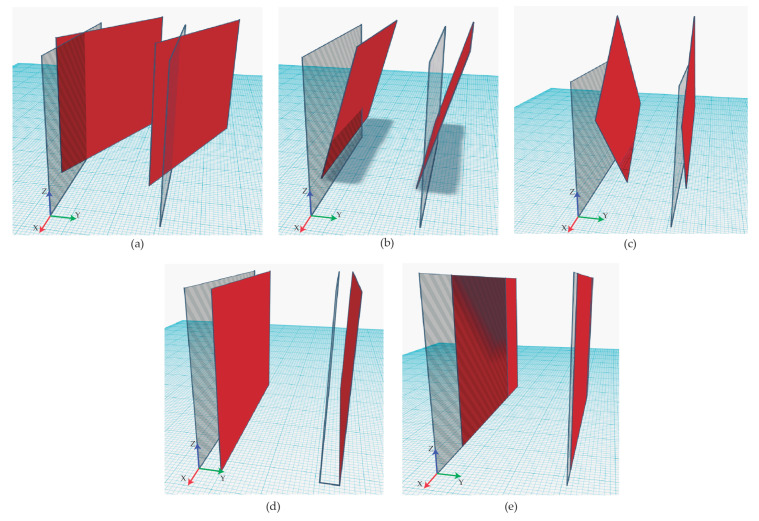
Visual representation of aligning errors between virtual planes (transparent) of the virtual 3D model and scanned planes (red) of the real world: (**a**) Z-axis rotation, (**b**) Y-axis rotation, (**c**) X-axis rotation, (**d**) Y-axis translation and (**e**) X-axis translation.

The goal of the validation process is to assess the displacement and inclination errors of the modeled planes by ARCore compared to their corresponding planes of virtual model. All possible cases are shown in Figure 14.

According to the following tests, rotation is only required around Z-axis (Figure 14a). Regarding the plane inclination, ARCore ensures that all modeled planes are fully vertical (Z axis), with normal vector (0,1,0), or completely horizontal, with normal vector (0,0,1). This condition has been validated calculating the dot product between the vector z=(0,0,1) and the normal vector of every detected plane and checking if the result is 0. That is true since the normal vector is always perpendicular to the vector z so the case Figure 14b is discarded, that is, ARCore always provides vertical planes when tracking vertical walls. According to the case of Figure 14c, it is not considered because this rotation does not change the normal vector of the plane. Consequently, we focus only on the rotation around Z-axis to study the error of plane inclination from real-world data. To measure this error, the angle (α) between the normal vector of the virtual plane and the normal vector of its corresponding plane, detected by ARCore, is calculated by applying the Equation (2).

(2) $$\alpha =\arccos(\frac{n_{1}n_{2}}{|n_{1}\left|\right|n_{2}|})$$

where $n_{1}$ is the normal of the virtual plane and $n_{2}$ is the normal of an ARCore plane.

Table 2 shows the average error regarding the six experiments carried out (1) using markers to estimate the initial orientation of the camera user and (2) without makers only considering inertial measurements of IMU’s device, that is, the compass. Our markerless-based approach requires that the 3D model of the building in the virtual world is orientated to the north. In this way, when the camera user is pointed to the north, the direction vector of the camera in the virtual world is aligned with the Y-axis according to our reference system in the virtual world. According to the results of the validation process, our method was focused on solving the rotation about the Z-axis by applying a rotation on virtual planes to be fully aligned with scanned planes from the real world. Thus, the angle between a virtual plane and its corresponding scanned plane from the real world is almost 0.

Additionally, we have checked ARCore accuracy in terms of distance in order to establish the level of confidence to estimate the position of the user camera in the virtual world. In this regard, distances between the user and detected walls of its surrounding environment are used to determine the real location of the virtual camera regarding the real world, as discussed in Section 2.4. Table 3 shows the difference between the real measurements made from one point in the test area to the five walls and their comparison with 16 measurements for each plane made with ARCore. As can be seen, the maximum error is 6 cm. However, the average error is less than 2 cm. This means that several measures provide distance values very similar to the real ones with less than 5 m away from the walls. This means that the situation in Figure 14d is discarded as a possible failure since the find errors were values under a few centimeters. Finally, displacement errors as the one depicted in Figure 14e are not important for an initial positioning if the user is located in the specific locations into the building, as discussed in Section 2.4. This mismatch should be considered during the navigation process, although the division of virtual walls into sections allows this adjustment to be carried out efficiently thanks to the topological connections between these sections. These error measures obtained during this validation process are incorporated into the algorithms and calculations described in Section 2.3 in order to define a confidence threshold when comparing both angles and distances.

Finally, the scanning process can be influenced by several factors of the user environment such as the lighting and occlusion. Nonideal scenarios with poor lighting and a high occlusion of walls by furniture pose a challenging recognition of the user position and orientation. Our method is only capable of capturing planar surfaces to provide a real-time and efficient processing of scanned data. Consequently, if the user environment is fully occupied by furniture, our method presents some limitations since the wall is occluded. Nevertheless, the topological segmentation carried out on the virtual model enables the relationship between sections of the wall, namely, if just a part of the wall is scanned, the proposed method is able to match this with its corresponding wall section of the virtual model. If no sections can be scanned, the user has to continue walking around the scenario in order to search any wall partially visible. According to those environments characterized by a poor lighting, our method presents some limitations since some walls cannot be detected from a far distance. Table 4 shows the accuracy of our method to set the user orientation and position considering both special factors of nonideal scenarios. The same area of experiments described before has been used. In general, most of the surrounding planes can be detected and matched with the virtual model with a almost similar accuracy to previous experiments. For nonideal cases, the real-time recognition of the user environment plays a key role but the user has to cover a larger area in order to capture a higher number of surrounding walls.

## 4. Results

According to the application of the previous methodology, the observed study scenario can be inspected using MR. After registering the user position and orientation and mapping the virtual 3D models on their correct locations, an interactive and intuitive visualization may be performed in real time. The proposed framework demonstrates the capabilities of smartphones to capture meaningful characteristics of the user environment and to display the shape of hidden facilities through mixed reality. These results emphasize the potential of our application to manage the status and monitor the deployment of indoor facilities onsite only using ubiquitous systems. In the following, the user navigation and the updating process are described. The functionality of this application may be also reviewed in the video attached to this manuscript as Supplementary Material.

### 4.1. Navigation Process

Navigation in the real world means to change the mobile camera position and orientation in real time. Therefore, if the physical camera is moving, this implies that the virtual camera must follow the same displacement. Both must be synchronized in order to maintain alignment.

Mobile motion is registered by the inertial measurement unit, providing an input data in meters per second squared (m/s^2^). Distance can be thus calculated according to this formulation, and then extrapolated to the virtual world. Apart from motion tracking, additional sensors such as the gyroscope are decisive to determine the visible infrastructures. The problem is that these registered values are not totally reliable when navigating through the scene. The solution is to take advantage of the spatiotemporal coherence from the accurate initial position by tracking real and virtual walls during the navigation process. Thus, each time that a new wall section is detected in the real world through ARCore, the twin wall of the virtual world is easily found between the candidates, as described in Section 2.3. This must coincide in distance and orientation with the one tracked. Then, topological relations connecting annexed walls sections facilitate the process. Following the user direction, it is possible to know in advance which are the following virtual walls. Using this method, small corrections are made in the virtual environment by comparing orientation and distances between virtual and scanned walls. This correctness is then maintained over time, facilitating access to infrastructure networks. Even in between hallways as depicted in Figure 11, navigation can be successful because wall sections, that are consecutive in the physical world, are also consecutive in the virtual world.

**Figure 15:**
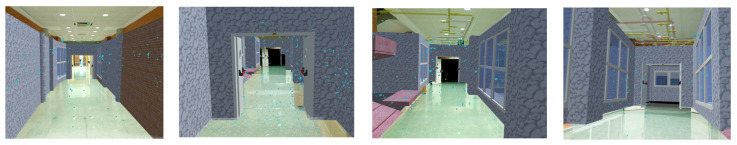
User navigation through a corridor of the study area.

Figure 15 shows some snapshots during navigation in the same university’s corridor from left to right. Walls are depicted to show alignment regarding physical walls, enabling mixed reality. This hall is divided by a couple of doors. The first one is crossed from the second to the third image. As the original 2D CAD project does not provide geometric information about wall heights, this value must be precalculated and assigned to all areas except those with stairs. Real heights of walls are not accurately assigned on purpose in order to visualize part of behind structures. The two right images of Figure 15 represent both virtual wall and infrastructure layers.

**Figure 16:**
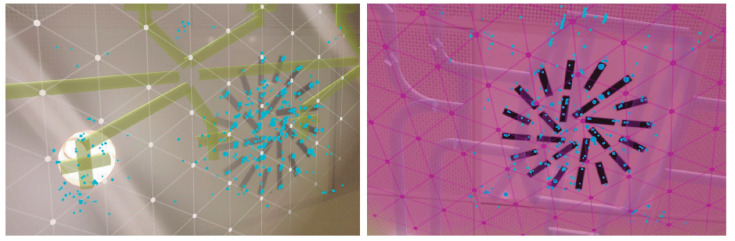
Nadir visualization of electrical and air-conditioning infrastructures.

For better accuracy in positioning regarding facilities embedded in the ceiling, the camera should look as perpendicular as possible, using the nadir plane. Figure 16 is an example, in which all the pipes associated with various infrastructures are depicted. We have checked it by visualizing the superposition of virtual facilities and the terminal elements of said infrastructure networks, such as lights or air conditioners.

### 4.2. Updating Process

**Figure 17:**
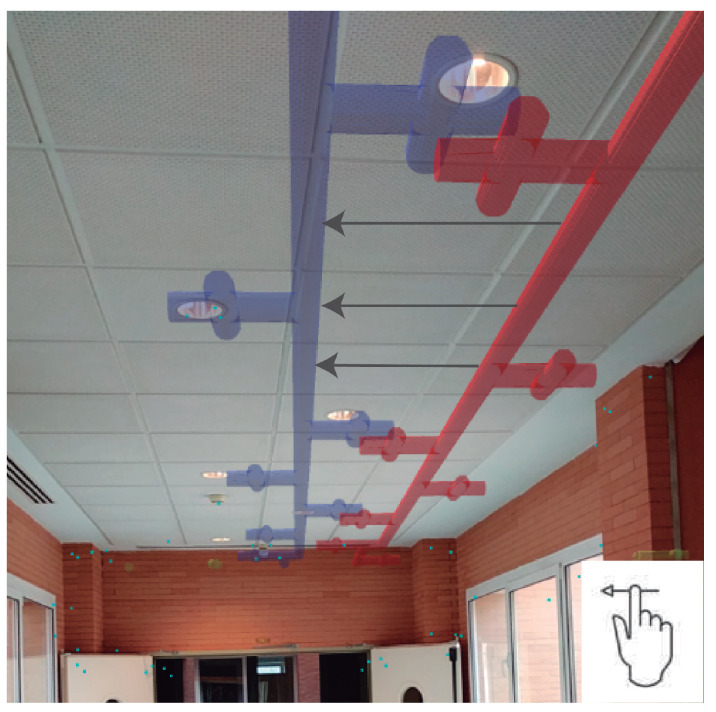
Updating process to change the position of a light wiring.

Even though we have not found inaccuracies in the input data, it is possible that reality and original plans show some kind of inconsistency. We have implemented the updated process, consisting of modifying some characteristics or aspects of the virtual model such as pipe type but also its geometry or spatial distribution. This modification may be necessary when it is verified that the physical model does not correspond to the virtual one, or perhaps after performing any infrastructure repair or update. This update process can be done directly on the mobile screen, through the Graphic User Interface (GUI), affecting the spatial arrangement of the items. In this way, several intuitive interactions can be performed using the proposed application. If the disposition of these facilities is affected, the first step is to select the specific facility. Selection is performed in the Unity environment, which implements the action over the geometry displayed in the scene. After that, the user displaces the selected item over the same wall plane, that is, only changes in coordinates X and Z. Therefore, this displacement can be done by dragging the selected element on the screen. Figure 17 shows both the red line located on an erroneous position and the blue wiring that represents the correct position after the updating process.

## 5. Conclusions

Mixed reality is a trending topic in a multidisciplinary research where novel applications coexist. Smartphones as ubiquitous systems to scan real-world data and process input data efficiently play a key role for launching mixed reality experiences. The development of new applications that take advantage of some of the sensors of smartphones for acquiring real-world data and multimodal data fusion are highly demanded in the fields of architecture and engineering. The current state of technology is quite mature and several APIs provide interesting features to capture information from the user environment. An example of this technology is Google’s platform ARCore that is integrated in the proposed application. We have observed a high precision of sensing data regarding the detection of the planes corresponding to walls, floors and ceilings in indoor scenarios. However, mobile sensing lacks accuracy in relation to camera positioning or orientation and many mixed reality applications share an important limitation to create augmented virtual objects on their correct position in the real world. This problem is overcome in this research from a geometrical perspective by capturing 3D planar surfaces of the user environment and comparing them with a virtual 3D model of the surveyed building.

In this paper we propose a disruptive application to be launched on the latest smartphones in order to visualize indoor infrastructures of buildings using mixed reality. This goal poses several challenges like the estimation of user position and orientation, the recognition of the user environment and the estimation of a geometric transformation to overlap virtual infrastructures on their correct locations. To this end, a novel methodology has been presented which contains some algorithms to overcome mentioned challenges. In this work, we have taken advantage of simple geometry which can be scanned from the user environment in order to estimate the user position and orientation in a virtual scenario which contains all CAD layers in 3D. In the preprocessing phase, topological relationships on the 3D geometry were established to generate wall sections that are a more feasible object to compare in real time with scanned 3D planes. During the real-time scanning, our method compares physical and virtual walls to estimate the user position and orientation and to correct the translation and rotation errors of virtual objects by applying a geometric transformation. To achieve this goal, some mathematical operations were carried out considering the normal vectors, distances and intersections of virtual and scanned 3D models. As a result, the user position and orientation are obtained and the virtual world is aligned thus, achieving a correct visualization of hidden infrastructure on walls and ceilings in mixed reality. Some guidelines have also been given in relation to the physical spaces where this technology is best adapted, for example, for well compartmentalized spaces.

The proposed method has been tested in indoor environments where walls are key features to determine the user position and orientation. Thus, augmented facilities can be visualized on a correct location using mixed reality. In addition, our solution is not too sensitive to furniture and partially occluded 3D planes may be recognized and scanned. As future research, we aim to use depth data in order to detect irregular surfaces and to also be able to understand the user environment characterized by special conditions like fire and poor lighting.

## Figures and Tables

**Table 1:** Formulation used to correct the user position in the virtual world.

Plane: parametric equation	$P_{A}=p_{1}+\lambda \vec{u}+\mu \vec{v}$, $\vec{u}=p_{2}-p_{1}$, $\vec{v}=p_{3}-p_{1}$
Plane: general form equation	Ax+By+Cz+D = 0, $\vec{n}=\mathrm{Ai}+\mathrm{Bj}+\mathrm{Ck}$
Distance: $p_{r}$ to plane $P_{A}$	$d_{\mathrm{rA}}=\frac{|{\mathrm{Ax}}_{r}+{\mathrm{By}}_{r}+{\mathrm{Cz}}_{r}+D|}{\sqrt{A+B+C}}$, $p_{r}=\left(x_{r},y_{r},z_{r}\right)$
Line:	$L_{A}=a+t\;\vec{\mathrm{ac}}$
$L_{A}^{\prime }$ parallel to $L_{A}$ at distance $d_{\mathrm{rA}}$	$L_{A^{\prime }}=a^{\prime }+t\;\vec{\mathrm{ac}}$ $a^{\prime }=a+d_{\mathrm{rA}}\vec{n},\;∥n∥=1$
Intersecting point:	$L_{A}=a+t\;\vec{\mathrm{ac}}$, $L_{B}=b+s\;\vec{\mathrm{bd}}$,
$p_{r}$ is $L_{A}$ in t	$s_{=}\frac{X_{\mathrm{bd}}Y_{\mathrm{ab}}-X_{\mathrm{ab}}Y_{\mathrm{bd}}}{X_{\mathrm{bd}}Y_{\mathrm{ac}}-X_{\mathrm{ac}}Y_{\mathrm{bd}}}$
$p_{r}$ is also $L_{B}$ in s	$X_{\mathrm{bd}}Y_{\mathrm{ac}}-X_{\mathrm{ac}}Y_{\mathrm{bd}}\neq 0$

**Table 2:** Mean error of experiments regarding the plane inclination on Z-axis.

Average Error	Plane 1	Plane 2	Plane 3	Plane 4	Plane 5
Using fiducial markers	0.5${}^{\circ }$	0.8${}^{\circ }$	0.3${}^{\circ }$	0.9${}^{\circ }$	0.1${}^{\circ }$
Without markers	10.9${}^{\circ }$	7.5${}^{\circ }$	11.4${}^{\circ }$	9.4${}^{\circ }$	8.5${}^{\circ }$
Our method	1${}^{\circ }$	0.3${}^{\circ }$	0.1${}^{\circ }$	0.5${}^{\circ }$	0.4${}^{\circ }$

**Table 3:** Validation of the user position based on distance measurements (m).

	Plane 1	Plane 2	Plane 3	Plane 4	Plane 5
Real distance	3.6	2.97	3.59	2.97	2.15
Our method:					
Average distance	3.59	2.95	3.57	2.97	2.12
Maximum error	0.06	0.04	0.05	0.06	0.03
Standard deviation	0.036	0.035	0.038	0.044	0.029

**Table 4:** Validation of the user position based on distance measurements (m) and the user orientation based on the alignment between virtual and scanned plane in a nonideal scenario.

	Plane 1	Plane 2	Plane 3	Plane 4	Plane 5
User position (distances)	3.69	Not detected	3.51	3.15	2.09
User orientation (angles)	1.2${}^{\circ }$	Not detected	0.11${}^{\circ }$	0.62${}^{\circ }$	0.45${}^{\circ }$

## Data Availability

Not applicable.

## Supplementary Materials

The following are available online at https://www.mdpi.com/1424-8220/21/4/1123/s1.
